# A Retrospect of the Progress of Microscopic Investigation, and of the More Important Recent Contributions to Normal and Pathological Histology

**Published:** 1850-11

**Authors:** Robert D. Lyons

**Affiliations:** Ex-clinical Assistant to the Meath Hospital, The Surgeons to the Anglesey Dispensary, Lecturer and demonstrator of Anatomy in the original School of Medicine, and Honorary Professor of Anatomy to the Royal Dublin Society


### RECORD OF MEDICAL SCIENCE.

### ANATOMY AND PHYSIOLOGY.

*A Retrospect of the progress of Microscopic Investigation, and of the more important recent Contributions to Normal and Pathological Histology.* By Robert D. Lyons, M. B., T. C. D., L. R. C. S. I. *Ex-clinical Assistant to the Meath Hospital, one of the Surgeons to the Anglesey Dispensary, Lecturer and demonstrator of Anatomy in the original School of Medicine, and Honorary Professor of Anatomy to the Royal Dublin Society.*

Of the several methods of investigation by which, during this, the first half of the nineteenth century, the science of medicine has been advanced, and so many important additions have been made to our knowledge as well of normal structure and function, as of the several lesions which are produced in both by disease, not one is entitled to more serious attention at the hands of the physiologist, the pathologist, and the practical physician, than that which reveals to us, by the aid of the microscope, the ultimate configuration and arrangement of the particles of the most complex tissue, and resolves into elements the most dissimilar what to the naked eye appears a homogeneous fluid.

While we acknowledge the deep debt of obligation which our science owes to the researches of the chemist, and fully appreciate the value of his labors, we cannot but think that the extensive and vigorous prosecution of microscopic investigation is destined to confer equally signal benefits on medicine. A retrospective glance at what has been already achieved in this department would appear sufficient to convince even the most sceptical; but it is to be regretted that an irrational conservatism, and opposition to what is new, with a profound veneration for all that the past has transmitted to us, are too often active and watchful to throw a barrier of hostile prejudice in the way of those who are bold enough to break new ground. We find that, even on the Continent, very recent writers have thought it necessary to enter at some length into the consideration of the claims which the microscope has on our attention, though neither amongst our French nor German neighbors can its application to scientific medical investigation be considered as new (*a*.)

And yet, if we are to believe in a gradual progress towards the perfection of our art, the new must be ever looked for, and its advent should be hailed with gladness; not, however, that it may supersede the old, as proclaimed by many of its too zealous advocates with an exaggerating enthusiasm, perhaps never wholly separable from the introduction of a new dogma; but that with new aids superadded to the means we are already in possession of, we may be the better able to undertake the investigation and solution of difficulties that have hitherto baffled our best energies. And who is there so confident in the powers of his art, that will venture to assert that it needs no amelioration, that it has reached its point of culmination? If there be such, let him reflect on the many occasions that the most scientific principles of diagnosis have failed to detect an existing lesion, that the most judiciously directed treatment has been unsuccessful; let him but look on the long category of nervous diseases, and sum up the amount of his knowledge of their causation and their pathology, or the value of his skill in their treatment.

Though our preface be apologetic, it can hardly be deemed superfluous, especially in the pages of an Irish periodical, issued from the Irish school were every department of medicine is represented, and cultivated with success, save one, and that one, as we firmly believe, destined to

(*a*.) Traite du Microscope, &c., par le Dr. Ch. Robin. 8vo. Paris, 1849, pp. 79, *et seq* The following passage is deserving of attention :—

"Il n'y a, comme on le voit, dans tout ce qui précè, rien de plus que ce que nous étudions dans les autres corps, ce sont les memês charactères, les mêmes propriétés; il n'y a de nouveau que le maniere des les observer, qui n'est elle même qu'une modification de nos moyens ordinaires d'observation appropiêes à leur petit volume.

exercise a powerful influence on our views of the most abstract as well as the most practical questions. We believe that we are justified in saying that no original communication of any importance has been made to histology, or any collateral branch of microscopic investigation, by an observer from the Irish school; though, as we are fully aware, many of our physicians and surgeons have occupied themselves in verifying the observations of others, and in no few instances have acquired practical skill in the employment of this instrument, and made useful applications of the results thus obtained in the practice of their profession (*a.*) It may, however, be anticipated, that before long the attention of the Irish school will be fully awakened to the importance of the subject, and that many will be found amongst us ready and willing to engage in this interesting department of medical investigation. Our object in the present retrospect has been as much to bring the subject prominently forward, as to give a succinct though brief account of some of the more remarkable contributions to histology, which have been recently made in the English and Continental schools. The limited space which can be afforded to communications like the present in a journal so fully occupied as this with subjects of a more directly practical nature, prevents us from giving as complete a resumè of the state of microscopical science as we could wish; it is to be hoped, however, that the several contributions to histology that we are about to bring under consideration will be found both interesting and important.

The systematic treatise of Quekett, which was brought under review in a former number of this Journal, enters so fully into the consideration of the mechanical arrangements of the microscope, that we feel it unnecessary to do more at present than refer to its pages such of our readers as may be anxious to learn any particulars of manipulation. A work of somewhat similar scope has been published (*b.*) by M. Chas. Robin, author of the treatise " Des Végétaux qui croissent sur l'Homme et les Animaux."

Though it is no part of our present intention to describe either the microscope itself or any of its accessory apparatus, we shall borrow from the treatise of the latter author some practical hints which may be of value to those who are actually engaged in making investigations. The experience of M. Robin is directly opposed to the opinion maintained by some, that the prosecution of microscopic investigation is injurious to the organs of vision, and he does not hesitate to say, "Depuis Leuwenhoëck, qui conserva d'excellents yeux jusque dans une extrême

(*a.*) It is well known to most of our readers that the late Dr. John Houston, of this city, occupied himself very assiduously at microscopic investigation. Drs. Carte, Aldridge, Hill, Moore, Steele, Fleming, &c. &c., possess good instruments and are excellent observers. The last-named gentleman made an interesting communication, on the subject of urinary deposits, to the Surgical Society, at its last meeting, and has on many occasions favored us with opportunities of examining the urine in several abnormal conditions.

(*b.*) Du Microscope et des Injections, par le Dr. Ch. Robin. 8 vo. Chez J. B. Baillière, à Paris, 1849.

vieillesse, tous ceux qui se sont beaucoup servis du microscope s'accordent sans exception à reconnaître que jamais ils n'ont ressenti le moindre trouble visuel." If we but reflect, as this author remarks further on, that the light of the microscope is, for the low powers, scarcely greater than that of the sky or a lamp, by reason of the small degree of concavity of the reflecting mirror, and that, as we raise the power of the object-glass, the amount and intensity of the light diminish proportionably, we see no reason why the habitual use of the microscope should tend to injure the visual apparatus. At all events, there is no ground for supposing that the eyes would be more liable to injury from this use of them, than from constant reading or writing, which are equally fatiguing occupations.

Amongst the embarrassments which the inexperienced microscopic observer meets with (they have occurred over and over again to the writer,) may be enumerated his mistaking for constituent parts of the object he is investigating, the several flaws, scratches, and innumerable imperfections of the ordinary glass slides, as also the presence of dust and extraneous matter. Occasionally, when the eye is brought very near the eye-glass during a protracted and careful examination, the movement of the lids throws the eye-lashes in front of the pupil, and they assume the appearance of large filaments crossing the field. But a source of error much more likely to escape detection will be found in the so-called *globules and filaments of the eye*, which do not depend on a too strong impression of the retina by the rays of light, as supposed by some, but will be found to exist in both eyes of every individual who looks through the microscope, being subject to certain varieties in different persons. We are indebted to M. Robin (*a.*) for a very satisfactory account of this phenomenon, of which we propose freely to avail ourselves. When we examine the field of the microscope, without placing an object in focus, a mass of small, perfectly round globules, all nearly equal in size, may be observed. They cover all parts of the field, except about a sixth externally, and a space somewhat smaller within. Two or three tortuous very pale filaments are seen a little external to the centre of the mass of the globules, a few of the latter adhering to them. The mass of globules is limited without by a flattened line of filament, somewhat brilliant in the centre, appearing of the size of a demi-millimetre, and either straight or slightly curved. Within it is limited by a filament more brilliant than the preceding, and remarkably flexuous, and folded on itself. *All the globules and filaments move together.* There appear to be two planes of globules, the one nearer to the eye with sharper outline; the other deeper, paler, more distant, and with their borders more undefined. These two planes sometimes move in opposite directions, but only through a small space, and quickly resume their places. They are in constant motion, and may be ascer-

(*a.*) This writer informs us that M. Donné, in his Cours de Microscopie, Paris, 1844, in 8vo., has given a very elaborate description of these globules and fibres. See also Atlas de Cours de Microscopie, Paris, 1845, in fol. pl. xx. fig. 83.

tained to obey the movements of the head of the observer, remaining stationary when the latter is fixed for an instant with both hands. The filament which limits the mass of globules externally can be brought to the centre of the field, and it may be then seen that there is a certain number of globules placed outside it. The internal tortuous filament can also be removed, and then external to it will be seen one or two other filaments, equally tortuous and brilliant, directed obliquely towards it. The globules are perfectly round, close to each other, and appear about a demi-millimetre in diameter. They present in the centre a brilliant point, surrounded by a dark, well-defined circle, which is itself surrounded by a second and last external concentric ring, as brilliant as the central point. Those of the deeper plane differ only in being less defined, some of them are in contact with, and impinge on one another, so that their dark borders touch. Besides these globules, some persons see others, larger and more transparent, which do not always occupy the same place in the field of vision. The external straight filament is brilliant in the centre, the borders being more obscure and less defined: it appears about one or one and a half millimetres in size, no globules adhere to it, and it is impossible to say whether it is hollow or full. The internal filament differs from the preceding only by its flexuosity, which causes it to occupy more space in breadth but less in length, and makes it more evident when brought to the centre of the field. The filaments placed amongst the globules are much more narrow than the preceding, they are two or three in number, tortuous, and about the length of a quarter of the field. They are not always so readily perceived as the larger ones, their borders being less marked, and less brilliant, at the same time that the globules encircle and appear to adhere to them. These globules are ordinarily disposed in pairs, and in contact one with another, each pair, however, being separated from the next by a certain interval.

It is unquestionably of the highest importance that we should be able to distinguish these little bodies from any part of the object we are examining; and, in general, no difficulty whatever will be experienced in doing so. When we consider that they follow the motions of the head, and are not affected by changing the positions of the glass slides, or by any movements communicated to the stage, we see at once that they must be referred to the eye of the observer. Hence the necessity on the part of the latter, of making himself thoroughly acquainted with their figure, position, and the peculiar modification which they may possibly be subject to in his own person. They have been, and may be, to many a source of painful apprehension and uneasiness, giving rise to the opinion that the organs of vision were threatened with disease. It cannot, therefore, be too generally made known, that the phenomenon is one experienced by all who make examinations with the microscope; that the conditions that produce it are universal; and that there is no reason for fearing that cataract, or amaurosis, will be the reward of patient and protracted microscopic investigation. As to the cause of these *globules and filaments of the eye* being seen under the microscope, much difference of opinion still exists. M. Robin appears to agree with

M. Donné in thinking, that it is in the liquor Morgagni we must look for their real seat; it would appear to us, however, more probable that they are due, as remarked by Wallace, to the filaments and globules which the investigations of Pacini, Treviranus, Hanover, and others, have proved to exist in the retina. We consider it quite possible that the posterior wall of the eye may reflect light, and thus give an image of itself, in the same manner that objects are seen in the bottom of a river through the water; that in fact the eye may perform the triple office of twice transmitting light, bringing it to a focus, and seeing it.

The work of Mr. Quekett contains a very excellent description of several varieties of micrometers, and gives judicious rules for the estimation, by their assistance, of the value of the magnifying powers of the different object-glasses.

The two processes hitherto most frequently in use on the Continent are, according to M. Robin, *the method of the camera lucida and double vision;* which consists in throwing, by means of a camera lucida, the magnified image of an objective micrometer (whose subdivisions are known) on a rule divided into millimetres, placed at the distance of distinct vision. By noting how many millimetres on the latter are covered by each hundredth of a millimetre of the micrometer, the magnifying power is thus found directly. The second process consists in looking with one eye at the subdivisions of an objective micrometer placed under the microscope, while with the other we regard the divisions of a rule or the points of a compass. The images of the two objects, painted separately on each eye, are thus superposed one on the other in the nervous centres; and with a little habit, it may be easily ascertained how many of the subdivisions of the rule are covered by a single division of the magnified micrometer.

Several sources of inaccuracy are stated by M. Robin to exist in both these methods; these he had detailed at length in his work(*a*); and he has accordingly proposed the following *method by the ocular micrometer*, which appears to answer all requirements, both as to simplicity and accuracy. It consists in employing an ocular micrometer, the superior glass of which magnifies exactly ten times. The micrometer placed in the focus of the superior glass of this ocular is one centimetre or half a centimetre, of which each millimetre is divided into ten parts. These tenths of a millimetre, being magnified ten times, equal each a millimetre, it is consequently a decimetre, or a demi-decimetre, each of whose subdivisions is equal to a millimetre, which is placed permanently in the ocular. Consequently, if an objective micrometer be placed under the microscope and that, in examining with the ocular, each hundredth of a millimetre of the first is magnified so as to cover three divisions of the second, we learn that the microscope magnifies 300 times.

The following method, which is but a slight modification of that proposed by M. Robin, has been adopted by the writer. It presents the advantage of great simplicity, and requires the use of only one micrometer, and with a little practice will enable the young microscopist to

(*a*) See his Treatise on the Microscope, pp. 134, 135, *et seq.*

make himself acquainted with the powers of the several object-glasses and eye-pieces he may chance to possess; a knowledge which it is the more necessary he should be able to procure for himself, as almost all the instruments in use and on sale in this city are unprovided with any scale or registered description of the powers of the several glasses. It will be necessary in all instances to possess an objective or stage micrometer, whose subdivisions are known; with this, an ordinary eye-piece, and one of the circular glass slips used for covering objects, we are in possession of abundant means for estimating the powers of the various object-glasses; and if a little care be used in the manipulation, a very considerable degree of accuracy may be obtained. The circular glass slip may be extemporaneously converted into an ocular micrometer, by marking off on it, from a rule, with a diamond, or a finely pointed pen, any of the smaller subdivisions of an inch. Thus marked, it may be passed into the eye-piece by unscrewing the ocular, and be allowed to rest on the stop or diaphgram; the ocular being now replaced, we proceed to ascertain its power, and the consequently increased dimensions of the divisions of the glass slip, which may be done in the ordinary way, by looking at them with one eye, while the other is thrown on the subdivisions of a rule. These interspaces, being large, are easily compared, and being superposed one on the other, as above described, the magnifying power of the ocular is thus obtained. If the objective micrometer be now brought into focus, we can easily see how many of its subdivisions correspond to one or more of the spaces which we have marked off, and the power of the object-glass is thus readily found.

For low power, and the comparison of tolerably large divisions of the micrometer and the rule, no difficulty will be experienced in ascertaining the magnifying number of a particular object or eye-glass, by the *method of superposition*, or that of looking with one eye through the microscope, and with the other at a rule, and at most only a few trials will be necessary in order to attain very considerable accuracy; but it will be found widely dIfferent when we come to deal with high powers and small divisions and hence the value of M. Robin's method, and the modification of it just proposed, in which, though the preliminary step is made by ascertaining the power of the ocular by a method of double vision, another and certainly a more accurate process is employed for estimating the power of the object-glasses, which necessitates the comparison of minute subdivisions, in which, of course, any error would be less likely of detection. As to any source of error which might possibly arise from placing a slip of glass between the ocular and field-glass of the eye-piece, it must be so very trifling as not to deserve any notice. By selecting a glass whose surfaces are neither convex nor concave, but as nearly as possible flat, the amount of deviation will be altogether inconsiderable.

By a reference to the tables given in the works of Robin and Quekuett, it will be seen that the number 800 represents the highest magnifying power which has been obtained for the object-glass by Nachet, a distinguished optician of Paris. In this no account is made of the influence of the eye-glass, which would, of course, add considerably to the power. While with the one-twelfth inch object-glass, and the eye-glass A, manufactured by Ross, of London, a magnifying power of 600 is obtained ; the same object-glass, giving with this maker's eye-glasses B and C, respectively, 870 and 1400, as the magnifying powers.

With powers far lower than these, however, many of the most useful and interesting investigations can be made. "The magnifying powers from 100 to 300," says M. Robin, "serve for studying the bones, the teeth the hairs, the hair-bulbs, and the glandular culs-de-sac, but only in what concerns their grouping in each *acinus:* the study of their epithelium demands higher object-glasses." We think, however, that this author had elsewhere over-estimated the value of the very high powers, and also the facility of recognizing by their aid, and distinguishing the several varieties of abnormal formation: "La prétendue impossibilité de distinguer les globules de pus, des globules blanc du sang des corpuscules du tubercule et une foule d'autres erreurs, tiennent à la même cause (les faibles grossisements)—p. 172.—*Dub. Quar. Jour.*

